# Fertility sparing surgery in malignant ovarian Germ cell tumor (MOGCT): 15 years experiences

**DOI:** 10.1186/s12905-021-01437-8

**Published:** 2021-08-04

**Authors:** Narges Zamani, Mohadese Rezaei Poor, Sedigheh Ghasemian Dizajmehr, Shima Alizadeh, Mitra Modares Gilani

**Affiliations:** 1 Department of Gynecologic Oncology, Vali-E-Asr Hospital, Tehran University of Medical Science, Tehran, Iran; 2 Department of Obstetrics and Gynecology, Niknafs Hospital, Rafsanjan University of Medical Science, Rafsanjan, Iran; 3 Department of Obstetrics and Gynecology, Faculty of Medicine, Urmia University of Medical Science, Urmia, Iran; 4 Department of Obstetrics and Gynecology, Vali-E-Asr Hospital, Tehran University of Medical Sciences, Keshavarz Avenue, Imam Complex, Tehran, Iran

**Keywords:** Fertility-sparing treatment, Malignant ovarian germ cell tumor, Reproductive outcome, Chemotherapy, Young adolescent

## Abstract

**Aim:**

We aim to evaluate the reproductive outcome of fertility-sparing surgery and chemotherapy among young women diagnosed with MOGCT of any stage.

**Methods:**

In the current retrospective study we evaluated 79 patients with malignant ovarian germ cell tumors (MOGCT) who visited at Imam Center, Vali-e-asr Hospital, Gynecologic Oncology department during 2001–2016. Reproductive outcomes (menstruation status and childbearing) followed fertility-preserving surgery and adjuvant chemotherapy by filling questionnaires. Statistical analysis was done with SPSS software, Chi-Square Tests were done, and significance was determined at *P* ≤ 0.05. Results among 79 young women who underwent fertility-sparing treatment, 72 patients followed up for reproductive outcome, and 7 patients excluded because of death (3 cases), XY genotyping (3 cases), and bilateral ovarian involvement (1 case). The mean age at presentation was 23 years. (Range: 19–33 years). The 5 and 10-year disease-free survival rate was 87% and 94.4%, respectively. The overall survival rate (OSR) was 94.4% at 5 and 10 years. Regular menstruation recovered in 60 of 72 patients after treatment (83%). All patients without adjuvant chemotherapy experienced regular menstruation, while normal menstruation was retrieved in 78% in the adjuvant chemotherapy group at the end of treatment. This retrieval of regular menstruation was not dependent on the age or number of chemotherapy cycles. 19 of 26 patients who attempted pregnancy were led to delivery (73%). No one required infertility treatments. The mean of chemotherapy cycles is related to a successful pregnancy.

**Conclusion:**

We showed patients with MOGCT could become pregnant and give birth if they desire. The advanced tumor stage wasn't the convincing factor for avoiding fertility preservation. Fertility sparing surgery with adjuvant chemotherapy is a safe treatment and results in a high fertility rate.

## Introduction

Malignant Ovarian Germ cell tumors (MOGCTs) are derived from primordial germ cells of the embryonic gonads. Ovarian Germ cell tumors account for 20 to 25% of all ovarian malignancies and the incidence of malignant MOGCT is 5% [1, 2]. Germ cell tumors are more likely to affect adolescents and women of reproductive age [3]. Except for dysgerminoma, for which the incidence of bilaterality is 10–15%, bilateral ovarian germ cell tumors are exceedingly rare [4]. Unilateral salpingo-oophorectomy with preservation of the contralateral ovary and uterus, combined with surgical staging, can be performed in most patients with MOGCT. Affecting young women, preservation of fertility is a major concern [5]. It is important to spare the contralateral ovary if possible in this young population [6]. Most adult women who were diagnosed with MOGCT are recommended to undergo adjuvant chemotherapy. The regimen of choice is bleomycin, etoposide, and cisplatin (BEP) [7–10].

Several series have documented normal reproductive function without compromising survival following fertility-sparing surgery and chemotherapy [11, 12]. Although impairment in ovarian function ovarian or premature ovarian failure is a risk of chemotherapy, most women who receive platinum-based therapy for three or four cycles retrieve regular ovarian function, and fertility is often spared in this group of patients [13–20]. The impact of platinum-based chemotherapy on adult women's ovarian function was described in a representative series of 71 patients treated with fertility-sparing surgery and combination chemotherapy (including cisplatin and bleomycin). Of these, 62 (87%) regained normal menstruation, and 24 of these women eventually had 37 offspring [19].

In this retrospective study, we aim to evaluate the outcome and safety of fertility-sparing surgery (FSS) and chemotherapy among premenopausal women diagnosed with MOGCT of any stage. We hypothesized that fertility-sparing surgery even with adjuvant chemotherapy is a safe treatment and results in a high fertility rate.

## Methods and materials

In the current retrospective study, we evaluated 79 patients with MOGCT who visited at Imam Center, vali-e-asr Hospital, Gynecologic Oncology department during 2001–2016. All patients were visited in the gyneco-oncology clinic and after documentation of the patient's history, physical exam, and para clinical findings, patients whom they suspected for MOGCT underwent FSS. Complete and Optimal FSS is defined as preserving the uterus and at least part of the contralateral ovary with no or < 1 cm residual tumor, respectively. Debulking and staging surgery were done by omentectomy and lymphadenectomy and peritoneal biopsy and cytology of peritoneal washing. Convincingly diagnosis was confirmed pathologically in all patients.

We collected data including age, chief complaint, time of surgery, pathology, grade, stage based on FIGO (International Federation of Gynecology and Obstetrics) classification, through inpatient case files. We also recorded information about adjuvant chemotherapy that the patients received after surgery.

We obtained additional data on MOGCT patients, by asking them to fill questionnaires on reproductive outcomes, menstruation status several months after treatment. We followed the patients by calling them or visiting in the clinic again. We collected fertility information (number of pregnancies and childbirth, childbearing desire, menstrual status, methods of pregnancy, gestational weeks at delivery, and obstetrical complication.

Statistical analysis was done with SPSS software v.20.0 *(SPSS Inc, Chicago, USA).* Chi-Square Tests were done, and significance was determined at *P* ≤ 0.05. Survival analysis was done using Kaplan–Meier method and compared using log‑rank test. Disease‑free survival (DFSR) was defined as the time from diagnosis to date of recurrence. Overall survival (OSR) was defined as the time from diagnosis to date of death or last follow‑up. Informed consent to participate in the study was obtained from all the patients. Ethical approval was obtained by the ethics committee of the Imam Khomeini Hospital Complex, Tehran University of Medical Sciences (TUMS) with the ethical code IR.TUMS.IKHC.REC.1397.256, and all methods were carried out following relevant guidelines and regulations.

## Results

We studied 79 young women who underwent fertility-sparing surgery, received adjuvant chemotherapy, and followed up in our clinic at the gynecologic oncology center, Imam Hospital during 2001- 2016. Seven patients were excluded because of death (3 cases), XY genotyping (3 cases), and bilateral ovarian involvement (1 case). The median follow-up time was 56 months (range: 8–194 months).

Table 1 summarizes patients' characteristics. The mean age at presentation was 23 years (range: 19–33 years). The main presenting symptoms were abdominal pain in 62 patients (86%) and abdominal distention was detected in 53 women (74%).

**Table 1:** Characteristics of patients who received fertility-sparing surgery

Median age, y (range)	
22 (19–33)	
*Main symptoms*	
Abdominal pain	62 (86%)
Abdominal distention	53 (74%)
*Pregnancy history before FST, n*	
Nullipara	52 (72%)
Multipara	20 (28%)
*Histological type*	
YST	9 (12.5%)
IMT	26 (36.1%)
DYS	37 (51.4%)
*FIGO stage*	
I	11 (15%)
II	23 (32%)
III	38 (53%)
*Adjuvant chemotherapy, n*	
Yes	60 (83%)

FST: Fertility sparing treatment; YST: yolk sac tumor; IMT: immature teratoma; DYS: dysgerminoma; FIGO: Internatinal Federation of Gynecology Obstetrics

A total of 72 patients confirmed for malignant ovarian germ cell tumor pathologically after resection, including dysgerminoma (51.4%), immature teratoma (36.1%), and yolk-sac tumor (12.5%). Complete and Optimal FSS is defined as preserving the uterus and at least part of the contralateral ovary with no or < 1 cm residual tumor, respectively. None of the patients underwent suboptimal surgery (> 1 cm tumor residue). Fortunately, we had done complete/optimal surgery (< 1 cm tumor residue) for all the patients. There was no survival difference with Complete and Optimal surgery in early or advanced stages. 72 MOGCT survivors who underwent FSS were finally assessed. Surgical staging according to the FIGO classified the patients in this manner: stage 1 (11 patients), stage 2 (23 patients), and stage 3 (38 patients). We advised most women to undergo adjuvant chemotherapy after surgery for MOGCTs, except patients with stage IA, grade 1 immature teratoma, and stage IA dysgerminoma because their outcomes are excellent following surgery alone. This group candidate for chemotherapy in the occurrence of recurrence. None of the patients chosen for neoadjuvant chemotherapy because of operable patients with good performance. 60 out of 72 patients received adjuvant chemotherapy with BEP for three to nine cycles (83%). Bleomycin was omitted for patients received over 3 cycles because of potential side effects (Table 1). The 5 and 10-year disease-free survival rate was 87% and 94.4%, respectively. The OSR was 94.4% at 5 and 10 years. 5–10 year DFSR and OSR of Stage I MOGCT patients were 100%. DFSR and OSR of Stage II–III MOGCT patients were 83% and 90%, respectively.

Regular menstruation recovered in 60 of 72 patients after treatment (83%). All patients without adjuvant chemotherapy experienced regular menstruation, while 47 among 60 patients (78%) in the adjuvant chemotherapy group had normal menstruation at the end of treatment. This retrieval of regular menstruation was not dependent on the age or number of chemotherapy cycles (Table 2).

**Table 2:** Relation of menstruation status with age and chemotherapy cycles

Menstruation status	Number	Chemotherapy cycles (mean)	*P* value	Age (mean)	*P* value
Regular	60 (83%)	3.10 ± 1.60	0.200	23.22 ± 4.98	0.419
Irregular	12 (17%)	3.69 ± 1.66		24.38 ± 5.35	

**Fig. 1:**
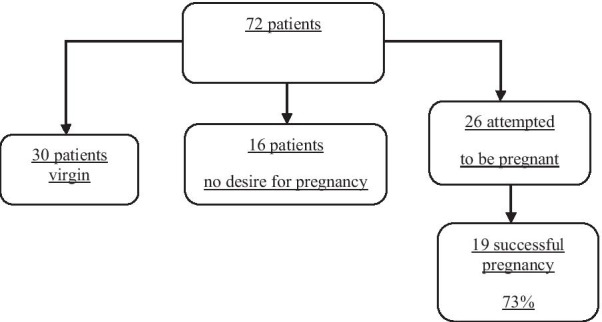
Patient distribution according to pregnancy desire

30 patients were virgins during follow-up and 16 patients had no desire for pregnancy, thus finally 26 attempted to be pregnant. Of these 26 patients, 19 had successful pregnancy lead to delivery (73%). The pregnancy rate was independent of age, stage, or type of pathology (Tables 3, 4). No one required infertility treatments (Fig. 1).

**Table 3:** Relation of pregnancy with age and chemotherapy cycles

Pregnancy desire (Total = 26)	Number	Chemotherapy cycles (mean)	*P* value	Age (mean)	*P* value
Pregnant	19 (73%)	2.79 ± 1.96	**0.044**	24.74 ± 3.22	0.262
Non-pregnant	7 (27%)	4.43 ± 0.78		26.57 ± 4.57	

Bold indicates a *p*-value less than 0.05 (typically ≤ 0.05) is statistically significant

**Table 4:** Relation of delivery with stages and histologic types

Histological type		Successful delivery
YST	9 (12.5%)	1
IMT	26 (36.1%)	9
DYS	37 (51.4%)	9
*FIGO stage*		
I	11 (15%)	3
II	23 (32%)	9
III	38 (53%)	7

YST: yolk sac tumor ; IMT: immature teratoma; DYS: dysgerminoma; FIGO: Internatinal Federation of Gynecology Obstetrics

The mean of chemotherapy cycles and age concerning successful pregnancy were shown in Table 3.

**Fig. 2:**
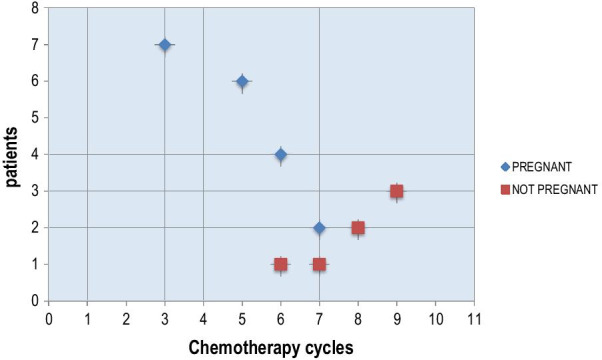
Scatter plot of chemotherapy cycles numbers and pregnancy

Figure 2 is a scatter plot showing the meaningful relationship between the number of chemotherapy cycles and pregnancy rate. Table 5 indicates reproductive results according to adjuvant chemotherapy (AC) cycle number.

**Table 5:** Reproductive results according to AC cycle number

Chemotherapy cycles numbers	Pregnant	Not-pregnant
3	7	0
5	6	0
6	4	1
7	2	1
8	0	2
9	0	3

## Discussion

MOGCT forms part of the gynecological malignancies, which involves almost always young women of reproductive age so, FSS is the most important and challenging fact for the patient and of course for the gyneco-oncologist [20]. Owing to the rarity of these tumors, data on efficacy are limited to single-arm clinical trials or retrospective series [21–27]. It has been proven radical surgery does not have survival advantage over conservative surgery despite the tendency to choose radical surgery due to life-threatening disease anxiety [28]. At least 80 percent of patients in early-stage disease receive Unilateral salpingo-oophorectomy with chemotherapy, resume normal menstrual function and, pregnancy without excess complications [18, 19]. There are limited reports on reproductive outcome in MOGCT, since the rarity of MOGCT and, just 30-year prognosis improvement experience following the BEP therapy [29]. In the present study, the reproductive function, including menstrual status and pregnancy rate evaluated in women treated between 2001 and 2016 in Gynecologic Oncology department, Imam Center Vali-e-Asr Hospital.

In previous studies, it was shown that FSS did not affect cancer prognosis in patients with MOGCT [30–34], but the pregnancy rate was not mentioned in most of them [29]. In this research, menstrual status, number of pregnancies and childbearing situation of the patients are studied.

Menstrual function and childbearing potential after FSS and BEP chemotherapy were evaluated in the study by Angiolo Gadduci et al. [29]. The study mentioned 50% of patients *become amenorrheic* while over 95% of them resume normal menses after treatment. The rate of successful pregnancy was not assessed. *25 years experience strengthened the principle of carrying out conservative surgery to preserve ovarian function and fertility for most young women with MOGCTs *[32, 33]*.* Current study represents 60 of 72 patient recovered regular menstruation, independent of age and number of chemotherapy cycles (Table 2).

In the Norwegian study conducted by Solheim [32] post-treatment fertility in 61 patients with MOGCT assessed by measuring Anti-Mullerian Hormone (AMH). The 15-year cumulative post-treatment fertility rate was 28% of survivors and the rate was higher in patients treated by 3 or less chemotherapy cycles.

In the analysis by Yang et al., on 106 patients showed 59 of them underwent FSS, 45 patients had normal menstruation [12]. 31 successful pregnancies occurred in 39 patients desired pregnancy. In another study by Nagoya University, 105 patients with MOGCT and underwent FSS were studied. They concluded that 42 of 45 patients, who desired childbearing, became pregnant and 56 babies were born [29].

Recently in 2019, Mikus et al. described a case series of 27 patients with MOGCTs treated with FSS and summarized 50% of patients actively tried to conceive became pregnant, with 12 deliveries [35].

This study was done in Iran due to high ratio of young women in the country population and, probability of MOGCT effect on fertility. At the end the result showed the optimal surgery leads to promising outcome for the patients. All the 72 patients underwent unilateral salpingo oophorectomy, optimal debulking, and staging surgery (omentectomy and lymphadenectomy and peritoneal biopsy and cytology of peritoneal washing) that was the standard management of the unilateral MOGCT. 60 patients received (3–9 cycles of) BEP chemotherapy. All the surgeries were done in Imam Hospital so, according to the identical methods applied in all operations, the final results are more credible.

The 30 patients were virgin and 16 patients had no desire for childbearing then, in 46 patients the fertility evaluation could not be run. It was one of our limitations because we could not evaluate the fertility rate in this group of patients who included 63% of our study population. On the other side, the 19 patients out of 26 (who attempted pregnancy) were led to delivery and all the neonates were well-being (Fig. 1). The pregnancy rate was independent of age, stage, or type of pathology but, it was seen the pregnancy rate improved by decreasing the chemo cycles (Tables 3, 4).

Gershenson et al. also have found in their study that the rate of pregnancy was higher in the patients with no chemotherapy or less than 3 cycles of chemotherapy. They also found a higher rate of fertility in younger patients and the patients with chemotherapies regimen without Cisplatin [31]. Solheim et al. reported the positive correlation between chemotherapy dosage and gonadal dysfunction [32].

The average age of the study population was relatively young, equals to the mean age of 23. This low age-mean may affect the fertility rate under question because of better ovarian reservoir provide higher fertility in young women. As all patients received the same chemotherapy regimen (BEP), there was no chance of evaluating the effect of drug on the fertility rate.

Some studies claimed that fertility-sparing surgery was the choice of treatment with ideal survival [11, 17, 33, 34, 36–38]. This research showed the preserving-fertility treatment may be the best management for young women with MOGCT to preserve fertility. The 5 and 10-year disease-free survival rate (DFSR) was 87% and 94.4%, respectively. Overall survival rate (OSR) was 94.4% at 5 and 10-year. The 5 and10-year DFSR and OSR of MOGCT Stage I patients were 100%. The DFSR and OSR of MOGCT Stage II–III patients were 83% and 90%, respectively. Survivorship was higher among stage I disease patients compared to stage II and III disease. Notably, this good fertility rate was achieved in the presence of chemotherapy as part of the treatment. Missing menstruation periods was common during chemotherapies sessions but, menstruation usually comes back to normal after completion of therapy.

Young women with MOGCT have a chance to preserve fertility by conservative surgery. Advanced stages should not be a contraindication to preserve the uterus and contralateral ovary. Surgical management and postoperative adjuvant platinum-based combination chemotherapy herald excellent prognosis.

This study showed patients with MOGCT could become pregnant and have childbirth if they wish. Despite the limitation of retrospective study, 76 patients with the rare Ovarian tumors managed in a single referral Gyneco-oncology center which had an Oncology team included: Gyneco oncologist, expert gynecologic pathologists, expert radiologist in the gynecologic field, and medical oncologist, furthermore the reproduction outcome of the patients followed through the department of reproductive and infertility of the center. In conclusion, Fertility-sparing surgery should consider even in patients with advanced-stage evidence. The results of the present study may encourage patients to overcome MOGCTs with FSS and give a definite way for gyneco-oncologist to do the best for young women.

## Data Availability

The datasets used and/or analyzed during the current study are available from the corresponding author on reasonable request.

## Abbreviations

MOGCT: Malignant ovarian germ cell tumor
BEP: Bleomycin, etoposide, and cisplatin
FSS: Fertility-sparing surgery
